# The Efficacy of the Three Types of Plaque Control Methods During Fixed Orthodontic Treatment: A Randomized Controlled Trial

**DOI:** 10.7759/cureus.38231

**Published:** 2023-04-27

**Authors:** Amit Kumar, Jaideep Singh, Pallavi Sinha, Vineet Vaman Kini, Harshal R Champaneri, Shashank Kumar Mishra, Anushree Tiwari, Ramanpal Singh

**Affiliations:** 1 Department of Orthodontics and Dentofacial Orthopaedics, Dr. B.R. Ambedkar Institute of Dental Sciences and Hospital, Patna, IND; 2 Department of Orthodontics and Dentofacial Orthopaedics, Maharana Pratap Dental College, Kanpur, IND; 3 Department of Oral Medicine and Radiology, New Horizon Dental College and Research Institute, Chhattisgarh, IND; 4 Department of Periodontics, MGM Dental College and Hospital, Navi Mumbai, IND; 5 Department of Periodontics, Vaidik Dental College and Research Centre, Daman, IND; 6 Department of Conservative Dentistry and Endodontics, Triveni Institute of Dental Sciences, Hospital and Research Center, Bilaspur, IND; 7 Clinical Quality and Value, American Academy of Orthopaedic Surgeons, Rosemont, USA

**Keywords:** chemical plaque control, orthodontics, dental plaque, fixed orthodontic therapy, plaque controlling agents

## Abstract

Background: When intraoral orthodontic devices are used, it becomes significantly more difficult to remove plaque effectively. Dentists and orthodontic specialists can come up with more effective preventive strategies while patients are undergoing fixed orthodontic work if they have a deeper understanding of the present scenario. In addition, individuals will become more aware of the importance of good dental hygiene habits as a result of this.

Objective: To assess and compare the effectiveness of a manual toothbrush, machine-driven toothbrush, and conventional mechanical toothbrush coupled with mouth rinse in removing plaque and maintaining gingival health in patients undergoing fixed orthodontic treatment.

Methods and materials: In this research, a total of 222 individuals who met the eligibility and exclusion requirements were randomly selected and offered their written consent. There were a total of 74 participants for each of the three different categories. Category A used a physically driven toothbrush. Category B used a motorized toothbrush. Category C used a physically driven toothbrush together with mouthwash containing 0.2% chlorhexidine gluconate. All study participants were assessed at baseline, one-month follow-up, and two-month follow-up to document the preliminary information, including that of the modified papillary bleeding index (MPBI) by Muhlemann, plaque index (PI) introduced by Silness and Loe, and gingival index (GI) introduced by Loe and Silness.

Results: In this study, the mean PI scores at the one-month and two-month follow-ups were minimum in Category C, while it was maximum in Category A at the two-month follow-up. The mean GI scores at the two-month follow-up were minimum in Category C, while it was maximum in Category A at the two-month follow-up. The mean MPBI scores at the two-month follow-up were minimum in Category C, while it was maximum in Category A. It was observed that participants in this trial who only used a typical mechanical brush experienced an increase in PI and GI scores after one and two months of follow-up. At the one-month and two-month follow-ups, it was noted that the values of PI, GI, and MPBI significantly decreased in the study participants using automated toothbrushes as well as in study participants using manual toothbrushes in conjunction with chlorhexidine mouthwash as compared to baseline values. However, when the three categories were compared, it was found that the research participants utilizing both a manual toothbrush and 0.2% chlorhexidine experienced the highest decreases in PI, GI, and MPBI values.

Conclusion: The reduction in the scores of PI, GI, and MPBI was maximum in orthodontic patients after two months when they apply manual toothbrushing along with 0.2% chlorhexidine.

## Introduction

Periodontal plaque is a biofilm that is both physically and physiologically structured. It is a group of microorganisms that are located on the surface of teeth as a coating and are encased in a framework of polymers that have both microbial and host origins [1]. Plaque on teeth has been defined as the soft, persistent substance present on the surface of the tooth that is difficult to remove with a simple water rinse [2]. Inflamed gums (gingivitis), which is characterized by erythema of marginal gingiva at the point where they meet the teeth, as well as minor edema and hemorrhage from the gingival border, is primarily brought on by dental plaque [3]. Personalized dental hygiene, which involves removing bacterial plaque from teeth as well as gingiva and preventing its re-agglomeration, is the preservation of mouth hygiene for the sustenance of proper oral health [4].

The value of maintaining good oral health relies on the individual's personal dental health, possessing the ability, lifestyle, motivation, education, oral hygiene training, and oral hygiene assistance [5]. Brushing your teeth is the most popular mechanical method of preventing plaque at home. There is strong evidence demonstrating that plaque, as well as gingivitis, can be most consistently controlled with teeth brushing as well as other mechanical cleaning techniques, assuming that washing is done thoroughly and at the right intervals [6]. Once intraoral orthodontic devices are used, efficient plaque clearance is noticeably hindered.

With a greater understanding of the existing situation, dentists and orthodontists can create more efficient preventive strategies while undergoing fixed orthodontic work. In addition, individuals will become more aware of the importance of good dental hygiene habits as a result of this. Therefore, the aim of the study was to assess and compare the effectiveness of a manual toothbrush, machine-driven toothbrush, and conventional mechanical toothbrush coupled with mouth rinse in removing plaque and maintaining gingival health in patients undergoing fixed orthodontic treatment.

## Materials and methods

This research was a concurrent arm, experimental, randomized, regulated, and observer investigation. It involved participants getting fixed orthodontic treatment who ranged in age from 13 to 35, as most orthodontic therapies were carried out in this age group.

**Figure 1:**
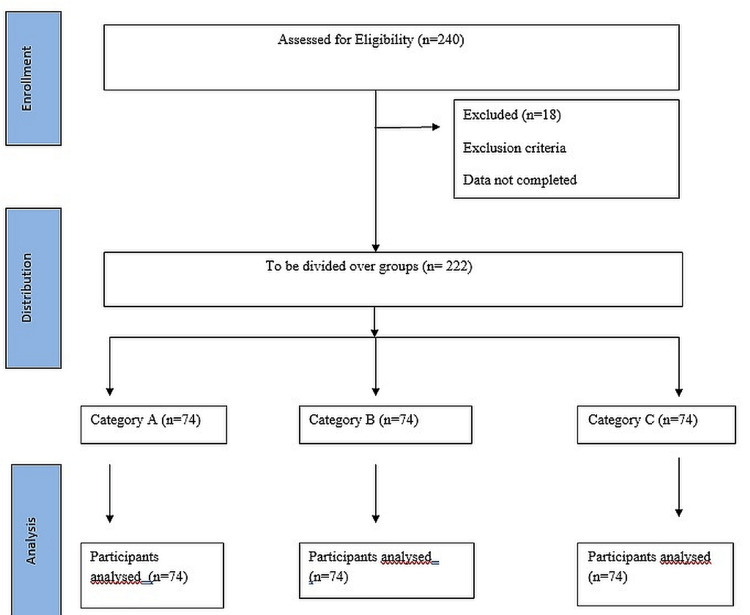
CONSORT flow diagram

The sample size was calculated using n = (z)2 p ( 1 - p ) / d2, where n = sample size, z = level of confidence according to the standard normal distribution (for a level of confidence of 95%, z = 1.67, p = estimated proportion of the population that presents the characteristic (when unknown we use p = 0.5), and d = tolerated margin of error (e.g., we want to know the real proportion within 5%). Using the above formula, a minimum sample size of 222 was calculated (Figure 1).

The total quantity of participants was determined by adding together all of the people on the waiting list for the outpatient clinic. As a result, 222 was chosen as the number of respondents to enable the homogenous distribution of 74 patients among the three experimental subgroups. All study participants who experienced concurrent complete maxillary arch as well as mandibular arch fixed orthodontic therapy employing MBT appliance (0.022 slots) and were righty, between 13 and 35 years old, willing to be involved with the research until its finalization, and did not have any systemic illnesses or medical conditions were enrolled in the research. The research excluded participants who were taking antibiotics, receiving lingual orthodontic work, or utilizing any additional plaque-eliminating tools, such as flossing or interproximal brushes.

**Figure 2:**
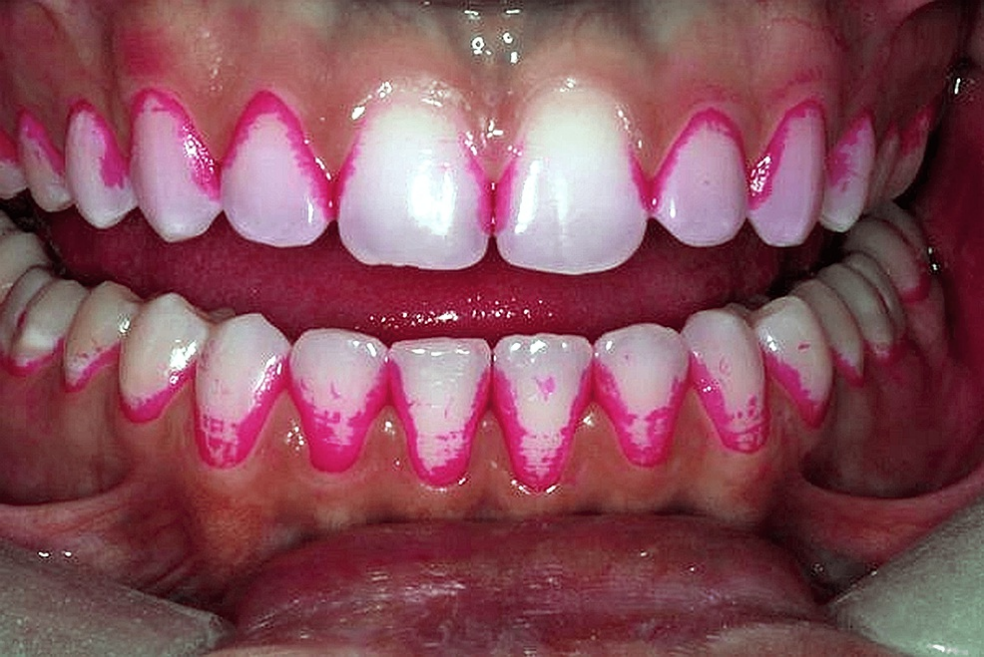
Plaque index scoring in patients

In this research, a total of 222 individuals who met the eligibility and exclusion requirements at Dr. B.R. Ambedkar Institute of Dental Sciences and Hospital were randomly selected and offered their written consent. The ethical clearance number is IEC/2022/11. A one-month flushing timespan was required after oral prophylaxis and the start of orthodontic therapy with fixed orthodontic appliances in order to cancel the effects of the ultrasonic scaler. All study participants were then called back one month later to document preliminary information, including that of the modified papillary bleeding index (MPBI), plaque index (PI) introduced by Silness and Loe, and gingival index (GI) introduced by Loe and Silness. When the investigator was calibrated, the average value of the kappa coefficient was determined to be 0.8, indicating a strong consensus. The intra-examiner heterogeneity was evaluated using the kappa variability analysis (Figure 2).

Following the acquisition of the preliminary information, the individuals were assigned randomly by using a simple method of randomization to one of the three intervention categories, namely, categories A, B, and C. The co-investigator, who was not participating in the assessment, provided labels for the commodities used in the experiment.

There were 71 participants in Category A, 73 participants in Category B, and 78 participants in Category C. Category A used a physically driven toothbrush. Category B used a motorized toothbrush. Category C used a physically driven toothbrush together with mouthwash containing 0.2% chlorhexidine gluconate. Since study participants 1, 3, and 2 in Category A, B, and C, respectively, didn’t show up for the follow-up, 70 participants in Category A, 70 participants in Category B, and 76 participants in Category C were analyzed finally.

During basepoint and every subsequent follow-up, the participants underwent oral hygiene guidelines and watched a demonstration of the right way to brush their teeth. After completing the oral hygiene program, the subjects were given a compliance worksheet to record each session. During the duration of the investigation, the participants also received periodic SMS notifications after every day. The individuals were then summoned back at one-month and two-month follow-ups, and an oral assessment was conducted in addition to noting the markers to evaluate the unintended effects like gingival recession in Category A participants and the unintended effects like bleeding gums and eroded enamel in Category B participants. Soft tissue assessment was done to look for any negative alterations brought on by mouthwash use and teeth staining in Category C.

## Results

The mean PI score at baseline in Category A was 1.3 ± 0.43. The mean PI score at baseline in Category B was 1.4 ± 0.57. The mean PI score at baseline in Category C was 1.4 ± 0.36. The difference in PI scores at baseline between the three categories was not statistically significant (p= 0.67). The mean GI score at baseline in Category A was 1.4 ± 0.48. The mean GI score at baseline in Category B was 1.5 ± 0.58. The mean GI score at baseline in Category C was 1.4 ± 0.45. The difference in GI scores at baseline between the three categories was not statistically significant (p= 0.84). The mean MPBI score at baseline in Category A was 1.2 ± 0.86. The mean MPBI score at baseline in Category B was 1.1 ± 0.33. The mean MPBI score at baseline in Category C was 1.2 ± 0.51. The difference in MPBI scores at baseline between the three categories was not statistically significant (p= 0.72).

The mean PI score at the one-month follow-up in Category A was 1.4 ± 0.47. The mean PI score at the one-month follow-up in Category B was 1.1 ± 0.52. The mean PI score at the one-month follow-up in Category C was 1.0 ± 0.47. The difference in PI scores at the one-month follow-up between the three categories was statistically significant (p= 0.031). The mean PI score was minimum in Category C, while it was maximum in Category A at the one-month follow-up. The mean GI score at the one-month follow-up in Category A was 1.6 ± 0.54. The mean GI score at the one-month follow-up in Category B was 1.1 ± 0.54. The mean GI score at the one-month follow-up in Category C was 0.9± 0.46. The difference in GI scores at the one-month follow-up between the three categories was statistically significant (p=0.026). The mean GI score was minimum in Category C, while it was maximum in Category A at the one-month follow-up. The mean MPBI score at the one-month follow-up in Category A was 1.0 ± 0.67. The mean MPBI score at the one-month follow-up in Category B was 0.7 ± 0.40. The mean MPBI score at the one-month follow-up in Category C was 0.6 ± 0.53. The difference in GI scores at the one-month follow-up between the three categories was statistically significant (p=0.001). The mean GI score was minimum in Category C, while it was maximum in Category A at the one-month follow-up.

The mean PI score at the two-month follow-up in Category A was 1.6 ± 0.46. The mean PI score at the two-month follow-up in Category B was 0.9 ± 0.47. The mean PI score at the two-month follow-up in Category C was 0.6 ± 0.40. The difference in PI scores at the two-month follow-up between the three categories was statistically significant (p=0.020). The mean PI score was minimum in Category C, while it was maximum in Category A at the two-month follow-up. The mean GI score at the two-month follow-up in Category A was 1.9 ± 0.30. The mean GI score at the two-month follow-up in Category B was 0.8 ± 0.43. The mean GI score at the two-month follow-up in Category C was 0.6 ± 0.48. The difference in GI scores at the two-month follow-up between the three categories was statistically significant (p=0.014). The mean GI score was minimum in Category C, while it was maximum in Category A at the two-month follow-up. The mean MPBI score at the two-month follow-up in Category A was 1.1 ± 0.51. The mean MPBI score at the two-month follow-up in Category B was 0.6 ± 0.52. The mean MPBI score at the two-month follow-up in Category C was 0.4 ± 0.41. The difference in MPBI scores at the two-month follow-up between the three categories was statistically significant (p=0.003). The mean MPBI score was minimum in Category C, while it was maximum in Category A at the two-month follow-up (Table 1).

**Table 1:** Comparing baseline, one-month, and two-month mean PI, GI, and MPBI values between the experimental categories PI: plaque index, GI: gingival index, MPBI: marginal papilla bleeding index

		Category A	Category B	Category C	p-value
Baseline	PI (mean ± SD)	1.3 ± 0.43	1.4 ± 0.57	1.4 ± 0.36	0.67
	GI (mean ± SD)	1.4 ± 0.48	1.5 ± 0.58	1.4 ± 0.45	0.84
	MPBI (mean ± SD)	1.2 ± 0.86	1.1 ± 0.33	1.2 ± 0.51	0.72
One month	PI (mean ± SD)	1.4 ± 0.47	1.1 ± 0.52	1.0 ± 0.47	0.031
	GI (mean ± SD)	1.6 ± 0.54	1.1 ± 0.54	0.9± 0.46	0.026
	MPBI (mean ± SD)	1.0 ± 0.67	0.7 ± 0.40	0.6 ± 0.53	0.001
Two months	PI (mean ± SD)	1.6 ± 0.46	0.9 ± 0.47	0.6 ± 0.40	0.020
	GI (mean ± SD)	1.9 ± 0.30	0.8 ± 0.43	0.6 ± 0.48	0.014
	MPBI (mean ± SD)	1.2 ± 0.51	0.6 ± 0.52	0.4 ± 0.41	0.003

When there was an intra-category comparison between the PI values at different time intervals in each category, then it was observed that the PI values in category A at baseline (1.3 ± 0.43), one-month follow-up (1.4 ± 0.47), and two-month follow-up (1.6 ± 0.46) increased as the time duration increased. The observations were found to have statistical significance (p=0.001). The PI values in Category B at baseline (1.4 ± 0.57), one-month follow-up (1.1 ± 0.52), and two-month follow-up (0.9 ± 0.47) decreased as the time duration increased. The observations were found to have statistical significance (p=0.001). The PI values in Category C at baseline (1.4 ± 0.36), one-month follow-up (1.0 ± 0.47), and two-month follow-up (0.6 ± 0.40) decreased as the time duration increased. The observations were found to have statistical significance (p=0.001).

When there was an intra-category comparison between the GI values at different time intervals in each category, then it was observed that the GI values in Category A at baseline (1.4 ± 0.48), one-month follow-up (1.6 ± 0.54), and two-month follow-up (1.9 ± 0.30) increased as the time duration increased. The observations were found to have statistical significance (p=0.001). The GI values in Category B at baseline (1.5 ± 0.58), one-month follow-up (1.1 ± 0.54), and two-month follow-up (0.8 ± 0.43) decreased as the time duration increased. The observations were found to have statistical significance (p=0.001). The GI values in Category C at baseline (1.4 ± 0.45), one-month follow-up (0.9± 0.46), and two-month follow-up (0.6 ± 0.48) decreased as the time duration increased. The observations were found to have statistical significance (p=0.001).

When there was an intra-category comparison between the MPBI values at different time intervals in each category, then it was observed that the MPBI values in Category A at baseline (1.2 ± 0.86), one-month follow-up (1.0 ± 0.67), and two-month follow-up (1.2 ± 0.51) had no statistical significance (p=0.231). The MPBI values in Category B at baseline (1.1 ± 0.33), one-month follow-up (0.7 ± 0.40), and two-month follow-up (0.6 ± 0.52) decreased as the time duration increased. The observations were found to have statistical significance (p=0.001). The MPBI values in Category C at baseline (1.2 ± 0.51), one-month follow-up (0.6 ± 0.53), and two-month follow-up (0.4 ± 0.41) decreased as the time duration increased. The observations were found to have statistical significance (p=0.001).

From these findings, it can be inferred that the PI, GI, and MPBI values decreased significantly in the study participants using an automated toothbrush as well as in the study participants using a manual toothbrush in conjunction with chlorhexidine mouthwash at the one-month and two-month follow-up as compared to the baseline values. However, when the three categories were compared, it was observed that the decrease in the PI, GI, and MPBI values was greatest in the study participants using manual toothbrushes along with 0.2% chlorhexidine.

## Discussion

While performing fixed orthodontic work, dentists and orthodontists can develop more effective preventive tactics for good oral health with a better awareness of the current situation [7-15]. In addition, as a result of this, people will be more conscious of the significance of excellent dental hygiene practices. In order to evaluate and compare the efficiency of a mechanical toothbrush, machine-driven toothbrush, and conventional mechanical toothbrush combined with mouthwash in eliminating plaque and maintaining gingival health, patients receiving fixed orthodontic treatment were enrolled in this study [16,17]. It was observed that participants in this trial who only used a typical mechanical brush experienced an increase in PI and GI scores after one and two months of follow-up. At the one-month and two-month follow-ups, it was noted that the PI, GI, and MPBI values significantly decreased in the study participants using automated toothbrushes as well as in the study participants using manual toothbrushes in conjunction with chlorhexidine mouthwash as compared to the baseline values. However, when the three categories were compared, it was found that the research participants utilizing both a manual toothbrush and 0.2% chlorhexidine experienced the highest decreases in PI, GI, and MPBI values.

The results are consistent with those of the research by Misra et al. [8], in which the control manual toothbrushing category experienced an increase in PI and GI scores but no improvement in plaque elimination or gingival well-being after 30 days. In contrast, the research by Hickman et al. [9] found that the conventional mechanical toothbrush cohort significantly reduced dental plaque from starting point to 30 days, with the marked improvement still evident at 2 months, and significantly reduced gingival inflammation from starting point to 30 days, with the alteration from baseline becoming non-significant by two months.

Periodontal plaque is a biofilm that is both physically and physiologically structured. It refers to the collection of microorganisms that coat the surface of teeth and are housed within a framework of polymers with microbial and host origins [18]. Plaque on teeth is characterized as a soft, lingering substance that is present on the tooth's surface and is challenging to eliminate with a simple water rinse [19,20]. Dental plaque is the main cause of inflamed gums (gingivitis), which are characterized by erythema of the marginal gingiva where it meets the teeth as well as mild edema and hemorrhage from the gingival border [21-23]. Personalized dental hygiene is the preservation of mouth hygiene for the sustenance of proper oral health. It entails eliminating bacterial plaque from teeth and gingiva, preventing re-agglomeration [24].

The PI and GI values increased in the study participants using manual toothbrushing. From these findings, it can be inferred that the PI, GI, and MPBI values decreased significantly in the study participants using automated toothbrushes as well as in the study participants using manual toothbrushes in conjunction with chlorhexidine mouthwash at the one-month and two-month follow-up compared with the baseline values. However, when the three categories were compared, it was observed that the decrease in the PI, GI, and MPBI values was greatest in the study participants using manual toothbrushes along with 0.2% chlorhexidine.

The study limitation was the small sample size and the short follow-up time.

## Conclusions

The importance of keeping good oral health depends on each person's specific dental health, abilities, lifestyle, motivation, knowledge, oral hygiene training, and support with oral hygiene. The decrease in the PI, GI, and MPBI scores was maximum in orthodontic patients after two months when they apply manual toothbrushing along with 0.2% chlorhexidine. Chlorhexidine can be used efficiently as an adjunct to routine scaling to avoid plaque formation in orthodontic patients.

## References

[1] Cobb CM (2008). Microbes, inflammation, scaling and root planing, and the periodontal condition. J Dent Hyg.

[2] Axelsson P, Nyström B, Lindhe J (2004). The long-term effect of a plaque control program on tooth mortality, caries and periodontal disease in adults. Results after 30 years of maintenance. J Clin Periodontol.

[3] Chapple IL, Van der Weijden F, Doerfer C (2015). Primary prevention of periodontitis: managing gingivitis. J Clin Periodontol.

[4] Figuero E, Herrera D, Tobías A, Serrano J, Roldán S, Escribano M, Martín C (2019). Efficacy of adjunctive anti-plaque chemical agents in managing gingivitis: a systematic review and network meta-analyses. J Clin Periodontol.

[5] Figuero E, Nóbrega DF, García-Gargallo M, Tenuta LM, Herrera D, Carvalho JC (2017). Mechanical and chemical plaque control in the simultaneous management of gingivitis and caries: a systematic review. J Clin Periodontol.

[6] van der Weijden GA, Hioe KP (2005). A systematic review of the effectiveness of self-performed mechanical plaque removal in adults with gingivitis using a manual toothbrush. J Clin Periodontol.

[7] Van der Weijden FA, Slot DE (2015). Efficacy of homecare regimens for mechanical plaque removal in managing gingivitis a meta review. J Clin Periodontol.

[8] Misra S, Pahwa N, Misra V, Raghav P, Singh S, Reddy M (2012). Maintaining periodontal health in patients undergoing orthodontic treatment. APOS Trends Orthod.

[9] Hickman J, Millett DT, Sander L, Brown E, Love J (2002). Powered vs manual tooth brushing in fixed appliance patients: a short term randomized clinical trial. Angle Orthod.

[10] Sim HY, Kim HS, Jung DU (2017). Association between orthodontic treatment and periodontal diseases: results from a national survey. Angle Orthod.

[11] Madariaga ACP, Bucci R, Rongo R, Simeon V, D’Antò V, Valletta R (2020). Impact of fixed orthodontic appliance and clear aligners on the periodontal health: a prospective clinical study. Dent J (Basel.

[12] Bollen AM (2008). Effects of malocclusions and orthodontics on periodontal health: evidence from a systematic review. J Dent Educ.

[13] Biria M, Jafary M (2015). A review on preventive measures and treatment of white spot lesions in patients with fixed orthodontic appliances. J Dent Sch Shahid Beheshti Univ Med Sci.

[14] Arici S, Alkan A, Arici N (2007). Comparison of different toothbrushing protocols in poor-toothbrushing orthodontic patients. Eur J Orthod.

[15] Khalaf K (2014). Factors affecting the formation, severity and location of white spot lesions during orthodontic treatment with fixed appliances. J Oral Maxillofac Res.

[16] Slot DE, Wiggelinkhuizen L, Rosema NA, Van der Weijden GA (2012). The efficacy of manual toothbrushes following a brushing exercise: a systematic review. Int J Dent Hyg.

[17] Beals D, Ngo T, Feng Y, Cook D, Grau DG, Weber DA (2000). Development and laboratory evaluation of a new toothbrush with a novel brush head design. Am J Dent.

[18] Sharma NC, Qaqish JG, Galustians HJ, Cugini M, Thompson MC, Warren PR (2005). Plaque removal efficacy and safety of the next generation of manual toothbrush with angled bristle technology: results from three comparative clinical studies. Am J Dent.

[19] Warren P, Thompson M, Cugini M (2007). Plaque removal efficacy of a novel manual toothbrush with MicroPulse bristles and an advanced split-head design. J Clin Dent.

[20] Moeintaghavi A, Sargolzaie N, Rostampour M, Sarvari S, Kargozar S, Gharaei S (2017). Comparison of three types of tooth brushes on plaque and gingival indices: a randomized clinical trial. Open Dent J.

[21] Marçal FF, Mota de Paulo JP, Barreto LG, de Carvalho Guerra LM, Silva PGB (2022). Effectiveness of orthodontic toothbrush versus conventional toothbrush on plaque and gingival index reduction: a systematic review and meta-analysis. Int J Dent Hyg.

[22] Yaacob M, Worthington HV, Deacon SA (2014). Powered versus manual toothbrushing for oral health. Cochrane Database Syst Rev.

[23] ElShehaby M, Mofti B, Montasser MA, Bearn D (2020). Powered vs manual tooth brushing in patients with fixed orthodontic appliances: a systematic review and meta-analysis. Am J Orthod Dentofacial Orthop.

[24] Al-Teen RM, Said KN, Abu Alhaija ES (2006). Siwak as a oral hygiene aid in patients with fixed orthodontic appliances. Int J Dent Hyg.

